# Self-stigma and depression among community-dwelling adults with physical disabilities in China: the chain mediating role of social participation and self-esteem

**DOI:** 10.3389/fpsyg.2026.1774111

**Published:** 2026-03-18

**Authors:** Qianqian Hu, Jingjing Gong, Aixiang Li, Rui Wang, Hui Chen

**Affiliations:** Linyi People's Hospital, Linyi, China

**Keywords:** community-dwelling persons with physical disabilities, depression, self-esteem, self-stigma, social participation

## Abstract

**Background:**

Against the backdrop of a rapidly aging population, the number of people with physical disabilities in China is on a steady rise. Physical disabilities contribute to the development of unique psychological characteristics among this group, seriously affecting their thoughts, behaviors, and quality of life. Therefore, this study aims to explore the action paths and predictive values of self-stigma, social participation, self-esteem, and their influence on depression among community-dwelling people with physical disabilities in China based on the modified labeling theory.

**Methods:**

A cross-sectional study was conducted from April to September 2023 among 280 community-dwelling persons with physical disabilities recruited from 8 counties and cities in Yanbian Prefecture, China. Validated multidimensional scales were used to measure depression, self-stigma, social participation, and self-esteem. SPSS 26.0 was employed for descriptive statistical analysis, and AMOS 26.0 was used to construct a structural equation model (SEM) and analyze the mediating effect.

**Results:**

The average score of depression was (40.94 ± 8.27). Pearson correlation analysis showed that self-stigma and social participation were significantly positively correlated with depression, while self-esteem was significantly negatively correlated with depression (all *p* < 0.05). Path analysis results indicated that self - stigma could directly affect depression (*β* = 0.297, *p* < 0.001), and also indirectly affect depression through social participation (*β* = 0.265, *p* < 0.01), self-esteem (*β* = 0.106, *p* < 0.01), and both social participation and self-esteem (*β* = 0.076, *p* < 0.01), with the proportions of these effects being of 39.92, 35.62, 14.25, and 10.22%, respectively.

**Conclusion:**

Social participation and self-esteem play a chain-mediating role between self-stigma and depression among community-dwelling people with physical disabilities. This offers a reference for developing social intervention strategies to improve the mental health of people with physical disabilities.

## 1 Introduction

Depression is a mood disorder characterized by persistent low mood and social withdrawal (Hankin, 2006), which impairs an individual’s cognition, behaviors, and quality of life. It manifests as sleep disturbances, decreased appetite, and may even lead to extreme thoughts and behaviors such as self-harm and suicide (Lee et al., 2024; Li et al., 2025). Surveys show that 40% of the 280,000 suicide cases that occur annually in China involve individuals suffering from depression (Wang, 2024). Population aging in China is accelerating, with the situation being most severe in the northeastern region. The number of persons with disabilities will also continue to rise, imposing a heavy burden on social development (Yang, 2025). Jilin Province, located in Northeast China, has a staggering 800,000 persons with disabilities, with the number of those with physical disabilities accounting for approximately half. The issues related to the physical and mental health of this population and their career development have attracted extensive attention from the Chinese government (China Disabled Persons' Federation, 2023). Persons with disabilities are disadvantaged in many aspects of social life. They often experience unfair social labeling and are regarded as a “mentally incompetent” or “disabled” group considered useless to society (Smith, 2025). In the modified labeling theory of Link (Hunter et al., 2017), it includes five core components Beliefs, Internalization of official labels, Response, Consequences, and Vulnerability. Beliefs refer to the awareness among people without a diagnosis of mental illness or addiction of the social and cultural stereotypes and expected devaluation toward their group. The internalization of official labels occurs when an individual is diagnosed with a mental health or substance use disorder. Based on these beliefs, they will be assigned corresponding labels, and over the course of their social experiences, they will gradually accept and internalize these labels as part of their self-cognition. Responses are the self-protective measures taken by individuals after experiencing label-related stigma. Consequences mean that after individuals take negative strategies, it may reduce their access to obtaining social support or other resources. Vulnerability refers to the fact that, due to reduced social support, individuals with mental illness or addiction may face an increased risk of worsening mental symptoms or relapse. The theory holds that once an individual is classified as a member of a disadvantaged group and anticipates being devalued or discriminated against, negative social perceptions increase the individual’s sensitivity to negative self-evaluation or rejection, triggering self-protective mechanisms and prompting some defensive coping behaviors (Glass et al., 2013; Poku et al., 2023). For example, they may avoid or withdraw from perceived as uncomfortable or threatening social interactions. This leads to lower income, a smaller social network, and a higher risk of recurrence or exacerbation of mental disorders (Salelew et al., 2025).

Influenced by stigma, persons with disabilities internalize these negative labels externally imposed as part of their self-evaluation, and form self-stigma through identification and acceptance. These negative stereotypes and labels expose them to unfair treatment such as prejudice and discrimination in aspects like daily life, employment, education, and medical care access (Kim et al., 2025). These experiences lead them to change their self-perception, increase their sense of frustration and shame in interpersonal interactions, and result in low self-esteem, reduced social participation willingness, and social isolation and depression (Chung and Lam, 2018).

Self-esteem is one of the core components of personality development and reflects an individual’s subjective self-evaluation. Previous studies have shown that self-esteem buffers depression, and its level affects an individual’s mental health, interpersonal relationships, personality integrity and stability, as well as subjective initiative (Chung and Lam, 2018). Social participation is an important indicator of public health. Individuals with low levels of social participation usually have a lower quality of life and poorer physical and mental health (Kim et al., 2024; Manxusa and Botha, 2025).

To date, there is a lack of research in China using the modified labeling theory to study the mental health of persons with physical disabilities, and social participation has not been included as an independent variable in related studies. The specific influence pathways and effect sizes among self-stigma, self-esteem, social participation, and depression remain unclear. Therefore, this study aims to explore the current level of depression among community-dwelling persons with physical disabilities based on the modified labeling theory, clarify the pathways among self-stigma (Internalization of official labels), social participation (response), self-esteem (consequence), and depression (vulnerability), and provide a basis for community workers and primary medical institutions to carry out targeted psychological interventions for community residents with disabilities, to improve their mental health.

### 1.1 Research hypotheses

*H1*: Self-stigma is positively correlated with depression.

*H2*: There is a negative correlation between social participation, self-esteem, and depression.

*H3*: There is a negative correlation between self-stigma and social participation, self-esteem.

*H4*: Social participation mediates the relationship between self-stigma and depression.

*H5*: Self-esteem mediates the relationship between self-stigma and depression.

*H6*: Social participation and self-esteem play a chain-mediating role in the relationship between self-stigma and depression.

## 2 Methods

### 2.1 Participants

A cross-sectional study was conducted using convenience sampling in eight counties and cities of Yanbian Prefecture, Jilin Province, China. From April to September 2023, a total of 280 eligible community-dwelling persons with physical disabilities were recruited.

Inclusion criteria: (1) Aged ≥18 years; with a health record at the community health service center; (2) Meeting the diagnostic and classification criteria for physical disabilities in the National Classification and Grading of Disabilities for Persons with Disabilities; (3) Clear consciousness, literacy, and normal communication ability; (4) Providing written informed consent and participating voluntarily in this study.

Exclusion criteria: (1) Severe hearing or visual impairment; (2) Being in the acute phase of a pre-existing disease or condition; (3) Having participated in a similar study in the past month.

### 2.2 Data collection

All researchers received standardized training before the study to ensure their full understanding of the survey process. First, researchers briefly explained the study purpose, procedures, and significance to the participants. After obtaining written informed consent, questionnaires were distributed on-site in the community, and participants completed them independently and anonymously. For participants who had difficulty understanding the items or had visual impairments, our trained researchers were responsible for providing standardized explanations of these items without any suggestive guidance. A total of 300 questionnaires were distributed. After excluding 20 invalid questionnaires, 280 valid questionnaires were recovered, with a valid response rate of 93.33%.

### 2.3 Assessment tools

#### 2.3.1 Demographic data questionnaire

A self-designed questionnaire was used, which includes items such as gender, age, ethnicity, residence, living status, educational level, marital status, monthly household income, personality, medical insurance type, underlying diseases, disability grade, duration of disability, activities of daily living (ADL), and types of social activities.

#### 2.3.2 Self-rating depression scale

The Self-Rating Depression Scale (SDS) was developed by Zung in 1965 and was subsequently revised by Wang et al. (2010). It consists of 20 items, including 8 items for somatic symptoms, 8 items for depressive mood, 2 items for emotional symptoms, and 2 items for psychomotor symptoms. The cutoff value of the standard score of SDS is 5. Scores 53–62 indicate mild depression, and scores 63–72 indicate moderate depression (Zung, 1965; Yao et al., 2025). In this study, the Cronbach’s α coefficient of this scale was 0.825, showing good internal consistency.

#### 2.3.3 Explanatory model interview catalog (EMIC) stigma scale

The Chinese version of the Explanatory Model Interview Catalog (EMIC) Stigma Scale by Chung and Lam (2018) is a unidimensional scale for assessing perceived stigma. It includes 15 items, and each item is rated on a 4-point scale. Except for Item 2, the scores for all items were scored: 3 = “Yes,” 2 points for “Maybe,” 1 point for “Uncertain,” and 0 points for “No.” The scores of the 15 items were summed. A higher total score indicated higher perceived stigma among the respondents. In this study, the Cronbach’sα coefficient of this scale was 0.831, showing good internal consistency.

#### 2.3.4 Self-esteem scale

The Rosenberg Self-Esteem Scale (RSES) was developed by Rosenberg in 1965 and later revised by the Chinese scholar Wang et al. (2010). It includes 10 items; items 3, 5, 8, 9, and 10 were reverse-scored. Tian (2006) suggested that, due to Item 8 in the original scale culturally inappropriate, it could be forward-scored or deleted. The scale used a 4-point Likert scale: 1 point for “strongly disagree,” 2 points for “disagree,” 3 points for “agree,” and 4 points for “strongly agree.” The total score ranges from 10 to 40, and a higher score indicates a higher level of self-esteem. In this study, the Cronbach’sα coefficient of the scale was 0.770, showing good internal consistency.

#### 2.3.5 Participation scale

Chung and Lam (2018) translated the Chinese version of the participation scale, which includes three dimensions: activity participation, social participation, and work-related participation. The scoring for each item is as follows: 1 point for no difficulty; 2 points for mild difficulty; 3 points for moderate difficulty; 4 points for severe difficulty. A higher total score indicated a lower level of participation. Based on the total score, five levels are classified: 0–12 points represent no significant limitation; 13–22 points represent mild limitation; 23–32 points represent moderate limitation; 33–52 points represent severe limitation; 53–90 points represent extreme limitation. In this study, the Cronbach’sα coefficient was 0.876, showing good internal consistency.

### 2.4 Statistical analysis

Descriptive statistical analysis was performed using SPSS 26.0. All the data in this study were normally distributed and were expressed as mean ± standard deviation ( $\bar{x}$ ± s). Pearson correlation analysis was used to examine the correlations among variables. AMOS 26.0 was used to construct a structural equation model (SEM) and test mediating effects. The SEM requires a sample size ≥200, and the variables are normally distributed. If the data do not conform to the normal distribution, data transformation or non-parametric methods need to be used. In the case of missing data, the maximum likelihood method is used to estimate the model parameters. The model fit criteria: χ^2^/df < 3, RMSEA < 0.08, GFI > 0.90, CFI > 0.90, AGFI > 0.90, IFI > 0.90, NFI > 0.90. If all the hypothesized model indices met the above standards, it indicates that the model fits well. If the 95% bias-corrected confidence interval (CI) based on 5,000 bootstrap samples did not include 0 and *p* < 0.05, the total effect, direct effect, and indirect effect were significant.

## 3 Results

### 3.1 Common method bias test

Harman’s single-factor test was used to examine the common method bias resulting from the self-report questionnaire. The results indicated that the explained variance of the first principal component was 25.06%, far below the critical value standard of 40%. This No significant common method bias, and subsequent data analysis was feasible.

### 3.2 Scores of depression, self-stigma, self-esteem, and social participation among physically disabled individuals in the community

Table 1 shows scores of depression, self-stigma, self-esteem, and social participation of community physically disabled individuals, as well as the scores of each dimension.

**Table 1:** Scores of depression, self-stigma, self-esteem, and social participation of community physically disabled individuals, as well as the scores of each dimension.

Variables	Range of values (points)	$\bar{x}$ ± s
Depression	0~60	40.94 ± 8.27
Somatic symptoms and psychomotor retardation	0~18	12.46 ± 2.65
Depressive affect	0~24	16.54 ± 3.66
Positive emotion	0~12	8.25 ± 1.89
Interpersonal relationship	2~8	3.69 ± 1.33
Self-stigma	0~45	25.71 ± 7.95
Participation	18~72	39.41 ± 11.76
Work-related participation	3~15	9.00 ± 3.16
Activity participation	7~35	14.60 ± 5.17
Social participation	8~40	16.26 ± 5.26
Self-esteem	10~40	22.44 ± 5.14

### 3.3 Differences in depression among community-dwelling persons with physical disabilities across demographic characteristics

In the demographic data, there were statistically significant differences in depression among community-dwelling persons with physical disabilities in terms of ethnicity, place of residence, educational level, monthly family income, personality type, medical insurance method, disability level, and self-care ability (*p* < 0.05), as shown in Table 2.

**Table 2:** Comparison of depression scores among community-based physically disabled individuals with different characteristics (*n* = 280, $\bar{x}$ *± S*).

Variable	Group	Score	*t/F*	*P*
Ethnicity	Han nationality	41.26 ± 9.39	0.031	0.001
	Other ethnicities	45.68 ± 8.93		
Residence	Rural area	46.30 + 9.22	0.202	0.002
	Urban area	41.85 + 9.33		
Educational level	Primary school or below	51.63 ± 8.60	18.785	<0.001
	Junior high school	45.01 ± 7.82		
	High school or vocational school	41.57 ± 8.83		
	College or bachelor’s degree	36.37 ± 8.55		
Monthly family income	< 1,000	45.46 ± 8.38	6.029	0.003
	1,000~ 3,000	41.50 ± 8.86		
	≥ 3,000	40.98 ± 11.74		
Personality type	Introverted	42.31 ± 8.62	7.178	0.001
	Extroverted	38.72 ± 8.21		
	Neutral	42.63 ± 7.02		
Medical insurance type	Residents’ medical insurance	43.26 ± 9.91	5.182	0.006
	Urban employee medical insurance	41.21 ± 8.68		
	Self-payment	48.91 ± 7.93		
Disability grade	Grade 1	48.88 ± 10.28	20.610	<0.001
	Grade 2	44.14 ± 6.47		
	Grade 3	39.78 ± 7.13		
	Grade 4	36.58 ± 8.49		
Self-care ability	Complete self-care	39.63 ± 8.40	5.683	0.004
	Partially self-care	41.74 ± 7.88		
	Unable to self-care	46.50 ± 7.66		

### 3.4 Correlation analysis of depression, self-stigma, self-esteem, and social participation among community-dwelling physically disabled individuals

Among community-dwelling physically disabled individuals, self-stigma was significantly and positively correlated with depression (*r* = 0.741, *p* < 0.01) and social participation (*r* = 0.728, *p* < 0.01), and significantly and negatively correlated with self-esteem (*r* = −0.601, *p* < 0.01). Self-esteem was significantly and negatively correlated with both social participation (*r* = −0.613, *p* < 0.01) and depression (*r* = −0.687, *p* < 0.01). Social participation was significantly and positively correlated with depression (*r* = 0.778, *p* < 0.01). See Table 3.

**Table 3:** Correlation analysis of self-stigma, social participation, self-esteem, and depression among community-living people with limb disabilities (*n* = 280, *r* value).

	Depression	Depressive mood	Positive mood	Physical symptoms and activity lethargy	Interpersonal relationships	Self-stigma	Participation	Activity participation	Social participation	Work-related participation	Self-esteem
Depression	1										
Depressive mood	0.927**	1									
Positive mood	0.850**	0.734**	1								
Physical symptoms and activity lethargy	0.883**	0.715**	0.674**	1							
Interpersonal relationships	0.687**	0.529**	0.487**	0.560**	1						
Self-stigma	0.703**	0.661**	0.579**	0.640**	0.444**	1					
Participation	0.658**	0.629**	0.577 **	0.582**	0.370 **	0.716**	1				
Activity participation	0.552**	0.531**	0.490**	0.485**	0.298 **	0.635**	0.906**	1			
Social participation	0.604**	0.563**	0.523 **	0.541**	0.375**	0.667**	0.894**	0.720**	1		
Work-related participation	0.482**	0.479**	0.424**	0.421**	0.231**	0.449**	0.658**	0.446**	0.400**	1	
Self-esteem	−0.659**	−0.630**	−0.577**	−0.578**	−0.381**	−0.694**	−0.640**	−0.566**	−0.564**	−0.456**	1

**p < 0.01.

### 3.5 Multivariate linear regression analysis of depression among community physically disabled individuals

Taking depression as the dependent variable, and the demographic data, self-stigma, self-esteem, and social participation that were significant in the univariate difference analysis as the independent variables, a multivariate linear regression analysis was performed. The results are presented in Table 4.

**Table 4:** Multiple linear regression analysis of depression among physically disabled people in the community.

Variable	*B*	*SE*	*β*	*t*	*p*
Constant	28.72	3.64	–	7.90	<0.001
Disability grade	1.43	0.40	0.14	3.60	<0.001
Self-stigma	0.31	0.06	0.27	5.05	<0.001
Self-esteem	−0.52	0.09	−0.28	−6.09	<0.001
Social participation	0.32	0.05	0.40	7.27	<0.001

R^2^ = 0.744, adjusted R^2^ = 0.727, F = 43.686, P < 0.05, “-” indicates no such data.

### 3.6 Mediating effect analysis and test

**Figure 1:**
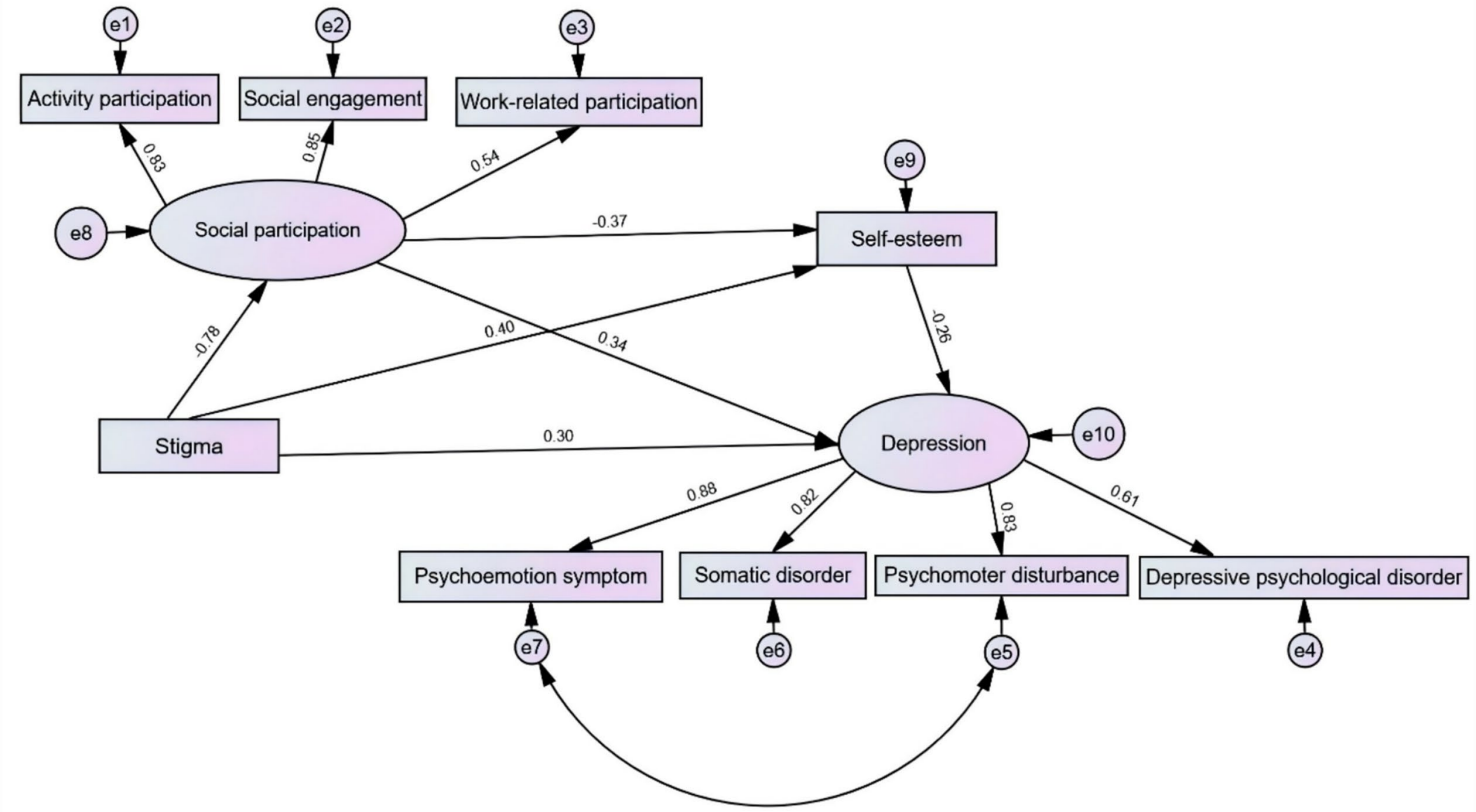
A chain mediating model of the impact of self-stigma on depression among community-based physically disabled individuals.

Using AMOS 26.0, a model diagram was established with depression as the dependent variable, self-stigma as the independent variable, and social participation and self-esteem as the mediating variables. Before the mediating analysis, the goodness-of-fit indices were evaluated. The results were as follows: χ^2^/df = 1.668, RMSEA = 0.049, GFI = 0.970, CFI = 0.990, AGFI = 0.941, NFI = 0.975, and IFI = 0.990. The model fit met the criteria. The results of the path analysis indicated that self-stigma had a positive predictive effect on depression (*β* = 0.297, *p* < 0.001); self-esteem had a negative predictive effect on depression (*β* = − 0.262, *p* < 0.001); social participation had a positive predictive effect on depression (*β* = 0.340, *p* < 0.001); self-stigma had a negative predictive effect on self-esteem (*β* = − 0.404, *p* < 0.001); self-stigma had a positive predictive effect on social participation (*β* = 0.781, *p* < 0.001); and self-esteem had a negative predictive effect on social participation (*β* = 0.273, *p* < 0.001). See Figure 1 for details. The Bootstrap method was used to further test the mediating effect. The original data were randomly sampled 5,000 times, and the 95% confidence intervals were calculated, respectively. The detailed results are presented in Table 5.

**Table 5:** Results of bootstrap mediation effect test.

Project	Estimate	SE	Z-value	Bias-corrected		Percentile		Effect size (%)
				95%CI		95%CI		
				Lower	Upper	Lower	Upper	
Indirect effect 1	0.265	0.094	2.820	0.106	0.482	0.106	0.480	35.62%
Indirect effect 2	0.106	0.035	3.029	0.044	0.186	0.039	0.178	14.25%
Indirect effect 3	0.076	0.028	2.714	0.033	0.147	0.030	0.139	10.22%
Total indirect effect	0.447	0.090	4.967	0.291	0.639	0.289	0.638	60.10%
Direct effect	0.297	0.099	3.000	0.087	0.476	0.083	0.474	39.92%
Total effect	0.744	0.034	21.882	0.671	0.806	0.671	0.806	─

Indirect Effect 1: Self-stigma → Social participation → Depression. Indirect Effect 2: Self-stigma → Self-esteem → Depression. Indirect Effect 3: Self-stigma → Social participation → Self-esteem → Depression.

## 4 Discussion

### 4.1 Analysis of the current situation and influencing factors of depression among community physically disabled persons

The results of this study showed that the depression score of physically disabled persons is (40.94 ± 8.27), which is at a moderately high level, indicating that their mental health status is poor. Introverted personality, inability to take care of oneself in daily life, low monthly family income, and high disability level were risk factors for depressive emotions among physically disabled persons, which was similar to previous studies (Inglis et al., 2024; Saleh et al., 2025). Introverted, physically disabled persons were unable to actively communicate with others to regulate their emotions or express their needs when facing stressful events. Therefore, they were more likely to develop depressive emotions compared with extroverted physically disabled persons. Compared with physically disabled persons living in rural areas, urban physically disabled persons with better family economic conditions were more likely to access high-quality medical resources and health education. Ensuring personal health is beneficial for reducing the incidence of depression (Pan and Hao, 2025). In addition, as a cultural resource, education level can increase individuals’ employment opportunities, improve their living standards, promote stronger self-control, and build a higher level of psychological security, thereby maintaining physical and mental health (Huang et al., 2025).

### 4.2 The relationship between self-stigma and depression

The findings of this study confirmed the hypothesis that self-stigma was positively correlated with depression. The self-stigma scores were at a moderately high level, which was similar to the research results of Zang et al. (2023). This may be attributed to the fact that persons with physical disabilities have experienced strange looks or differential treatment from others in social life. They perceived that the outside world labels them as “outsiders” and holds a stereotypical view of them as a vulnerable group. They tend to view themselves through others’ judgments, evaluate their self-worth in a distorted way, and reduce their self-value, forming incorrect self-perception, accompanied by a sense of shame (Cai, 2023). Self-stigma could seriously damage the social functions of persons with physical disabilities. When they experienced a sense of stigma, they would hold a negative attitude towards themselves and engage in behaviors deviating from the social group. They might even feel disgusted with themselves, complain about the unfairness of the world, and hate their disabled state (Montesano et al., 2025). Subsequently, they would isolate and seclude themselves, avoid social interactions, be reluctant to have close contact with others, care more about the views and evaluations of the outside world, fear that others would know and discuss their physical disabilities, and experience increased psychological pressure, and this psychological stress had a more significant correlation with loneliness and depressive emotion (Montesano et al., 2025; Song et al., 2025).

### 4.3 The mediating role of social participation between self-stigma and depression

The results of this study indicated that social participation plays a significant mediating role between self-stigma and depression. Social participation acts as a bridge linking individuals to society and is an important indicator of public health. Participation restriction was positively associated with depression level. Proactive social participation helps individuals foster a healthy personality, satisfy their psychological needs, achieve self-value, and enhance life satisfaction. Such individuals tend to adopt a more adaptive cognitive attribution style when confronted with setbacks. Their degree of internalization of stigma and social alienation was relatively lower, and their severity of depressive symptoms was also less severe (Lu et al., 2022). Therefore, community workers should provide platforms and opportunities for social participation to community-dwelling persons with physical disabilities, encourage them to actively integrate into society, help them establish life goals, make them feel needed and capable of contributing, and recognize their social value. By doing so, this can enhance their sense of self-worth and social responsibility and alleviate negative emotions.

### 4.4 The mediating role of self-esteem between self-stigma and depression

This study confirmed that self-esteem plays a significant mediating role between self-stigma and depression. In terror management theory, self-esteem is regarded as a buffer against depression. The level of self-esteem influences an individual’s mental health, interpersonal relationships, personality stability and integrity, as well as subjective initiative. High self-esteem was linked to healthy and socially acceptable behaviors, while low self-esteem was inconsistent with socially normative cognition and behavior (Greenberg et al., 1997). Such individuals tend to adopt positive coping strategies to maintain their self-worth when encountering negative evaluations from the outside world or stressful events, and they often experience lower depression levels. On the contrary, those with low self-esteem were more sensitive to external opinions, and their stress coping patterns were significantly associated with the high incidence of depression (Lasalvia et al., 2025). Therefore, community workers should provide targeted support for persons with physical disabilities, help them establish positive self-esteem and cognition, guide the physically disabled group to cope with setbacks proactively, overcome psychological distress, reduce the generation of negative emotions, and maintain physical and mental health.

### 4.5 The chain mediating role of social participation and self-esteem between self-stigma and depression

This study revealed the underlying mechanism between self-stigma and depression among community-dwelling individuals with physical disabilities, and further verified the chain mediating path consisting of social participation and self-esteem. While expanding the application scope of the labeling theory, it also provided a more comprehensive social perspective for understanding the mental health challenges of this special population. In addition, it was found that social participation was significantly correlated with self-esteem, which indicated that individuals with physical disabilities can break both physical and psychological isolation by enhancing their social engagement, gradually reduce the sense of inferiority caused by stigma perception, and effectively prevent the occurrence and spread of negative emotions. Previous studies had shown that social participation can expand social networks and access to social resources, and meet individuals’ psychological dependence needs. This was in line with the social contagion theory, which stated that by enhancing social integration, individuals’ physical and mental development can be promoted, thereby improving social adaptation, stimulating the social responsibility of individuals with physical disabilities, and enhancing self-esteem. This was not only closely associated with the reduction of stigma internalization and the alleviation of negative emotions, but also closely related to the individual’s mental health development and the improvement of quality of life (Zuckerman et al., 2021; Zhang et al., 2025). These findings suggest that we should change the previous simplistic support model for individuals with physical disabilities, develop a targeted intervention system focusing on the social network and precise mental health management of individuals with physical disabilities, empower this group, and create a social environment for equal participation to improve their subjective well-being and life confidence.

### 4.6 Practical significance

Based on the revised labeling theory, this study accurately captured the unique psychological experiences of individuals with physical disabilities in the processes of social labeling. It provided a novel theoretical perspective for analyzing the underlying mechanism of the impact of self-stigma on depression. Simultaneously, it provided strong theoretical support and practical guidance for subsequent targeted and scientific mental health interventions. Community workers and rehabilitation personnel should regularly assess whether this group shows signs of self-stigma. At the cognitive level, they should actively guide and assist them in developing a positive attitude toward disability.

We should create a “de-labeling” social interaction space for them. For example, organize activities such as social gatherings, sports meets, and various interesting competitions to encourage their active participation. Help them break away from the shackles of stigma, rebuild self-identity through social participation, and enhance psychological resilience by enhancing self-esteem. Ultimately, we can achieve a mutual promotion of individual mental health and social civilization, demonstrating the humanistic warmth and practical depth of the social support system.

### 4.7 Limitations

Firstly, due to the particularity of the population, the subjects of this study were only the physically disabled individuals in the Yanbian Prefecture of China. Their social culture, economic level, health care services, and ethnic disability cognition all exhibit certain regional particularities, so the sample may not fully represent the overall characteristics of the physically disabled individuals in the community-dwelling persons with physical disabilities across China. The research conclusion was restricted when extrapolated to similar groups in other regions of our country. It is suggested that future research should be conducted in multiple regions across the country to enrich the sample diversity and improve the representativeness and generalizability of the research conclusion. Additionally, cross-sectional studies do not allow causal inference. The relationships between self-stigma, social participation, self-esteem, and depression might change dynamically over time, thus showing temporal changes. The self-report questionnaire format may be limited by factors such as response bias and subjective cognitive deviation, which may affect the measurement of stigma and depression, and potentially affect the objectivity and reliability of the research results. Future longitudinal studies should be conducted to track the influence of latent variables on the depression of community physically disabled individuals at different time periods and the long-term mechanism, reducing self-report bias and improving the reliability of the research results, to offer more comprehensive and scientific evidence-based support for the mental health intervention of community physically disabled individuals.

## 5 Conclusion

This study provided a comprehensive exploration of the mechanism through which self-stigma influenced depression among community-dwelling individuals with physical disabilities, based on the mediating model proposed by the modified labeling theory. It confirmed that self-esteem and social participation play a significant chained mediating role between self-stigma and depression. Therefore, community workers should not only provide routine physical care of these individuals but also pay attention to their psychological well-being. It is also necessary to advocate to the public to respect their equal rights to development, promote and build a “diverse, inclusive, equal, and friendly” social ecosystem. Through the combined efforts of external support and internal empowerment, the goal of promoting the physical and mental health of this group can be achieved.

## Data Availability

The original contributions presented in the study are included in the article/supplementary material, further inquiries can be directed to the corresponding author.
